# Inter-observer reliability in transect-based observations of environmental waste in greater accra and kisumu: implications for waste management

**DOI:** 10.1007/s13762-024-05625-5

**Published:** 2024-04-22

**Authors:** J. Okotto-Okotto, M. Dzodzomenyo, L. Okotto, P. J. Shaw, S. Damkjaer, G. A. Myers-Hansen, E. E. Boafor, J. Wright

**Affiliations:** 1 Victoria Institute for Research On Environment and Development (VIRED) International, Off Nairobi Road, P.O. Box 6423-40103, Rabuor, Kenya; 2 Ghana School of Public Health, University of Ghana, P.O. Box LG 13, Legon, Accra, Ghana; 3 School of Spatial Planning and Natural Resource Management, Jaramogi Oginga Odinga University of Science and Technology, P.O. Box 210—40601, Bondo, Kenya; 4 School of Geography and Environmental Science, University of Southampton, Highfield, Southampton, SO17 1BJ UK

**Keywords:** Inter-rater reliability, Municipal waste, Sub-Saharan Africa, Survey methods

## Abstract

Transect surveys are widely used to quantify mismanaged plastic waste discarded to the environment. However, very few studies have quantified inter-observer reliability in environmental waste surveys. The aim of this study was to assess inter-observer reliability for a set of environmental waste indicators derived from transect surveys in slum areas of two sub-Saharan African cities. Pairs of observers independently recorded counts of scattered waste items, large waste piles, and burnt waste along 64 environmental transects in Kisumu, Kenya, and Greater Accra, Ghana. Concordance correlation coefficients were used to measure inter-observer reliability for derived indicators measuring mismanaged waste density, waste composition, waste origins of policy concern, and waste disposal practices. Evidence across all observers consistently showed that single-use disposable diapers, discarded Personal Protective Equipment, and bagged or bottled water are locally important constituents of mismanaged waste in both cities that should be addressed through urban waste management strategies. In both cities, there was generally excellent inter-observer reliability for density of burnt waste and density of large waste piles (concordance correlation coefficient > 0.9 for all but one observer pair), but weak to moderate inter-observer reliability for scattered waste density and densities of specific waste items such as disposable nappies and discarded Personal Protective Equipment. Our study shows that beach litter survey protocols can be adapted for use in slums. However, to generate robust estimates of scattered waste, its composition, and waste origins of policy concern in slums, assessment of inter-observer reliability should be incorporated into field team training.

**Supplementary Information:**

The online version contains supplementary material available at 10.1007/s13762-024-05625-5.

## Introduction

Actions are needed to improve global use of materials resources, reduce waste, and gain fuller value from waste materials. Target 12.4 of the United Nations’ Sustainable Development Goal 12 aims to “… achieve the environmentally sound management of chemicals and all wastes throughout their life cycle …" (United Nations 2022). Many waste components are hazardous to the environment or human health, including waste pharmaceutical and personal care products such as perfluoroalkyl substances and endocrine disruptors (Chaturvedi et al. 2021). However, our study focuses particularly on mismanaged plastic and plastic composite waste, which can be deposited on sea-beds, be ingested by and entangle marine fauna (Wright et al. 2013), then ultimately be absorbed by marine fauna and by humans (Schwabl et al. 2019). Coastal mega-cities are a concern as a plastic waste source, many of which are located in developing countries and face complex issues in relation to municipal waste management (Adedara et al. 2023). Such cities are forecast to be significant sources of plastics entering the oceans (Jambeck et al. 2015). Waste collection and management facilities and infrastructure are limited or absent in many developing countries (Njoku et al. 2015; Adedara et al. 2023), where mismanaged waste released into the environment—particularly burnt or dumped waste (Nagpure 2019)—presents notable challenges. Failure to collect and treat municipal waste in an appropriate manner presents direct and indirect risks to human health (Giusti 2009), loss of potentially recoverable materials (ten Brink et al. 2018), and risks to the proximate (Ayeleru et al. 2020) and global (Galgani et al. 2019; Jambeck et al. 2015) environment.

Analysis of international trade databases indicates that the African continent imported 172 million tonnes of plastics and polymers between 1990 and 2017, with an additional 15 million tonnes of plastic produced within Africa (Babayemi et al. 2019). Temporal analysis of trade data for six African countries showed increasing plastic/polymer imports in all countries (Babayemi et al. 2019). Given this background, the United Nations Environment Programme (UNEP) plastic pollution hot-spotting framework (Boucher et al. 2020) provides a basis for evidence-based action. This framework identifies five hot-spot types: application hot-spots, products whose consumption leads to significant mismanaged plastic waste; geographic hot-spots, areas where mismanaged waste proliferates; polymer hot-spots, specific plastic polymers disproportionately contributing to pollution; waste management hot-spots, points in waste management flows where plastics leak into the environment; and sector hot-spots (e.g. agricultural, domestic, or industrial generation of mismanaged plastic).

Numerous methods exist for quantifying mismanaged waste in the environment and providing evidence to inform plastic hot-spotting. Environmental transect surveys, for example, have been widely used by research teams or citizen scientists to record waste along randomly located transects. Transect surveys to quantify beach litter are well established (van Gool et al. 2021; Smith and Markic 2013), and both the National Oceanographic and Atmospheric Organisation (NOAA) (Opfer et al. 2012) and UNEP (Cheshire et al. 2009) provide guidelines for their implementation. Urban environmental transect surveys have likewise been used to quantify standing waste densities and fluxes (Ryan et al. 2020), illegal waste dumping (Nagpure 2019), waste related to the COVID-19 pandemic (Okuku et al. 2021; Ammendolia et al. 2021), and waste burning (Nagpure et al. 2015; Das et al. 2018). Other means of quantifying mismanaged waste in the environment include classification of high spatial resolution imagery from satellites (Georganos et al. 2021), unmanned aerial vehicles (UAVs) (Kako et al. 2020; Youme et al. 2021), and Google StreetView (GSV) (Rzotkiewicz et al. 2018). However, earth observation analysis requires further development before it can be implemented at scale (Georganos et al. 2021) and inter-observer agreement in GSV-derived waste metrics interpretation is typically only moderate (Mooney et al. 2014; Marco et al. 2017). Field surveys are thus likely to remain an important means of quantifying mismanaged waste in the environment for the immediate future.

Despite widespread use of transect surveys, inter-observer reliability in relation to such transect surveys remains unclear. Few studies have considered the potential for inter-observer disagreement when recording waste items and there is thus little insight into the potential for such surveys to yield reliable, actionable information. A large-scale Australian beach litter study used multiple observers to reduce inter-observer disagreement impacts on data reliability (Willis et al. 2017), but otherwise, few studies have explicitly addressed inter-observer agreement. Where resources allow, UNEP guidelines for beach litter surveys recommend the resurvey of a sample of transects by a field supervisor (Cheshire et al. 2009), whilst the US National Marine Debris Monitoring Program requires a deviation of no more than 20% in duplicate waste count observations following such resurvey (Sheavly 2007). We hypothesise that inter-observer agreement in the challenging field environment of slums will be lower than for beach surveys, but sufficient to characterise mismanaged waste.

Given the potential value of data relating to mismanaged, scattered waste in the environment (e.g. Rodseth et al. 2022) for policy and planning (Rodseth et al. 2020), it is important to understand how variation between observers influences the reliability of data thus acquired. The objective of this study is therefore to assess inter-observer reliability in urban environmental transect surveys of waste and its consequent impacts on urban environmental waste indicators. Given particular concerns over slums lacking waste collection services as potential future sources of mismanaged plastic waste (Jambeck et al. 2015), the study focuses on slums in two sub-Saharan African cities, Kisumu in Kenya, and Greater Accra in Ghana.

### Study sites

Fieldwork was undertaken in Kisumu, Kenya, and Greater Accra, Ghana. These two countries were chosen because of (1) their contrasting plastic waste management policies and (2) the presence of slums lacking waste services. Kenya is among several East African countries that have banned plastic bags, instead promoting reusable ‘Uhuru’ bags (Behuria 2021). In contrast, Ghana is among several West African countries where a large packaged water industry has emerged (Stoler 2012), with 58% of urban households consuming sachet water (water sold in 500mL plastic sleeves) as their main drinking water source in 2019 (Ghana Statistical Services and ICF, 2020b). Kisumu city’s population was 398,000 in 2019 (Capuano Mascarenhas et al. 2021), at which time over 60% of its residents lived in slums lacking adequate access to water, sanitation, and waste services (Sibanda et al. 2017). Only an estimated 20% to 35% of 200 to 450 tonnes per day of domestic waste is collected in Kisumu (Capuano Mascarenhas et al. 2021). In contrast, among Greater Accra’s population of 5.0 million in 2021 (Ghana Statistical Services 2021), 51% of households had waste collection services in 2010 (Ghana Statistical Services 2013). The city of Accra’s population generates an estimated 1552 tonnes per day of domestic waste (Miezah et al. 2015). The three sub-counties comprising Kisumu cover 387km^2^, whilst Greater Accra region covers 2,767 km^2^, excluding more rural Ga South and West districts. At district level, Greater Accra’s maximum population density is 33,600 people per km^2^ (Ghana Statistical Services 2021), whilst Kisumu’s most densely populated sub-county has 4,700 people per km^2^ (Kenya National Bureau of Statistics 2019). In Greater Accra, fieldwork took place from 31st August to 19th October 2021. In Kisumu, fieldwork took place from 10th September to 17th November 2021.

## Materials and methods

### Sample design

In each city, 30 and 32 urban Enumeration Areas (EAs) were selected based on probability-proportional-to-size sampling, based on population census data for 2010 in Greater Accra and 2009 in Kisumu, respectively. This sample was designed to estimate intra-class correlation (ICC), which is an inter-observer agreement measure. Assuming an ICC of 0.6 for scattered waste density, 8 surveyors, a desired 95% ICC confidence interval width of 0.2, alpha of 0.05, and power of 0.8, it was estimated that 60 transects (two per EA) would be required (Bonett 2002). Eligible EAs were those that met one or more of the UN Habitat criteria for a slum. UN Habitat considers an area to be a slum where the majority of households live in over-crowded housing, have unimproved sanitation, unimproved water sources, lack secure tenure, or lived in housing of non-durable construction (Lopez Moreno 2003). In this study, lack of waste collection services was included as an additional slum criterion. EAs dominated by communal establishments were also excluded. EAs that did not meet these criteria were excluded following initial area reconnaissance.

### Field team composition and training

The Kisumu survey team consisted of eight graduates and students, two with environmental or planning postgraduate diplomas, recruited to reflect typical research survey team composition. Individual team members’ field survey experiences varied from two to 20 years. Subject specialists provided intensive two-day training, which initially covered principles of waste identification, establishment of unbiased transect lines, sampling points along transects, quadrat placement and sampling, observation, mobile data entry, community liaison, and related ethical issues. A subsequent day of practical demonstrations took place in neighbourhoods meeting inclusion criteria but not selected for the main study, followed by a pre-testing exercise. More experienced team members were randomly paired with less experienced colleagues; then, each pair was randomly allocated EAs to survey. Training in Greater Accra followed a similar process, with a survey team of ten staff, five with postgraduate degrees in public health-related disciplines, with the remainder holding graduate degrees in other disciplines. Team members, who had three to 13 years’ research experience, were randomly allocated into two groups, comprising a supervisor and two random surveyor pairs. Each group was then randomly allocated EAs.

### Fieldwork implementation

Survey CTO software (Dobility Inc., 2021) was used to record all observations directly onto tablet devices, and non-differential Global Positioning System (GPS) receivers on tablets were used to record survey locations and aid navigation. In each EA, two publicly accessible transect routes were identified running perpendicular to one another, representative of the wider EA. Where an EA was crossed by a drainage line such as a stream or urban storm drain, this formed one of the transects selected for the survey. To measure waste fluxes and not solely standing waste stocks, transect surveys were repeated at different times on the same day. In Kisumu, field teams surveyed each transect in the morning, lunchtime, and evening, with a different team surveying the transect at lunchtime, all following the same protocol. Due to logistical constraints in the larger conurbation of Greater Accra, teams surveyed transects there in the morning and early evening only.

On each visit, transects were surveyed separately but simultaneously by two individuals, without conferring. Surveyors first recorded the number and composition of scattered waste items within a two-metre radius, approximately every 50 m (measured in paces, adjusting for individual stride length) along the transect. Since fieldwork took place during the coronavirus disease 2019 (COVID-19) pandemic, waste items were counted but not collected and weighed to minimise infection risks to survey teams. For international comparability, broad waste composition categories followed those used by the World Bank (Kaza et al. 2018), with more detailed categories used for plastics and Water, Sanitation, and Hygiene (WASH)-related items, including personal protective equipment (PPE). Teams then re-walked the transect, recording locations of all large (i.e. greater than 1m in diameter) waste piles. Two quadrats were randomly placed on each large waste pile and surveyors estimated the number and composition of waste items on each pile’s surface. Finally, surveyors walked along the transect a third time, recording all locations of burnt waste.

In Kisumu, two transects in 32 EAs were initially surveyed by two staff on one occasion, resulting in 128 sets of transect observations. Subsequently, 31 of these 32 EAs were surveyed by two staff at three times of day (morning, lunchtime, evening; Sect. "Fieldwork implementation"), giving a further 372 observations. This gave a total of 500 transect observations. In total, eight Kenyan staff worked in four pairs.

In Greater Accra, because of staffing difficulties, two of 30 EAs were surveyed by only one observer and were excluded from analysis. For the remaining 28 EAs, two observers each surveyed every selected transect twice (morning and evening; Sect. "Fieldwork implementation"). In 23 of these EAs, two transects were surveyed as planned, generating 184 transect observations. Among the remaining EAs, three transects were surveyed in two EAs, five transects in two further EAs, and 12 transects in a final EA, generating a further 110 observations. All the 294 observations in Greater Accra were retained for analysis, but inversely weighted each EA by the number of transects surveyed when comparing cities. In total, ten Ghanaian staff worked in six pairs.

### Analysis

Transect-level indicators of environmental waste, expressed as densities per unit area surveyed, were constructed from survey data within four domains: total mismanaged waste; waste composition; waste disposal practices; and waste origins of policy concern. Total mismanaged waste indicators comprised total scattered waste density and large waste pile density. Burnt waste pile density was calculated as a waste disposal indicator, whilst the proportion of scattered plastics was calculated as a waste compositional indicator. Indicators reflecting origins of policy concern comprised discarded Personal Protective Equipment (PPE) density due to the surge in its production, consumption, and disposal during the COVID-19 pandemic (Ammendolia et al. 2021); disposable nappy (diaper) density, due to the paucity of evidence relating to child faeces disposal as solid waste (Bain and Luyendijk 2015); and water sachet or bottle density, a particular concern in Ghana where packaged water is widely consumed (Stoler 2012).

To measure inter-observer agreement, Bland and Altman plots were examined for each observer pair with at least five shared transect observations using the Stata version 15.0 *blandaltman* package, first examining the distribution of inter-observer differences. A 20% difference has been proposed as acceptable limits of agreement for environmental waste surveys (Sheavly 2007), and in these initial plots, it was found that inter-observer differences increased significantly with the magnitude of waste densities. Percentage rather than absolute differences were therefore used to compute Bland and Altman limits of agreement and bias (mean percentage difference) from these plots, with confidence limits. Lin’s concordance correlation coefficient was calculated and related statistics (Lin 1989) for mismanaged waste indicators using the Stata version 15.0 *concord* package. Since scattered waste observations were made approximately every 50 m, to quantify spatial variation in waste indicators within transects, transect-level ICCs were also calculated for scattered waste indicators via an unconditional means multi-level regression model (Peugh 2010). Finally, to assess how inter-observer disagreement could affect waste indicator comparisons between Kisumu and Greater Accra, one observer’s data were randomly selected per transect. Robust regression (White 1982) was then used to test for transect-level differences in waste indicators between Kisumu and Greater Accra, whilst accounting for clustering of observations within EAs. This test was then repeated using data from the second set of observers.

## Results and discussion

### Summary of waste indicators

**Fig. 1:**
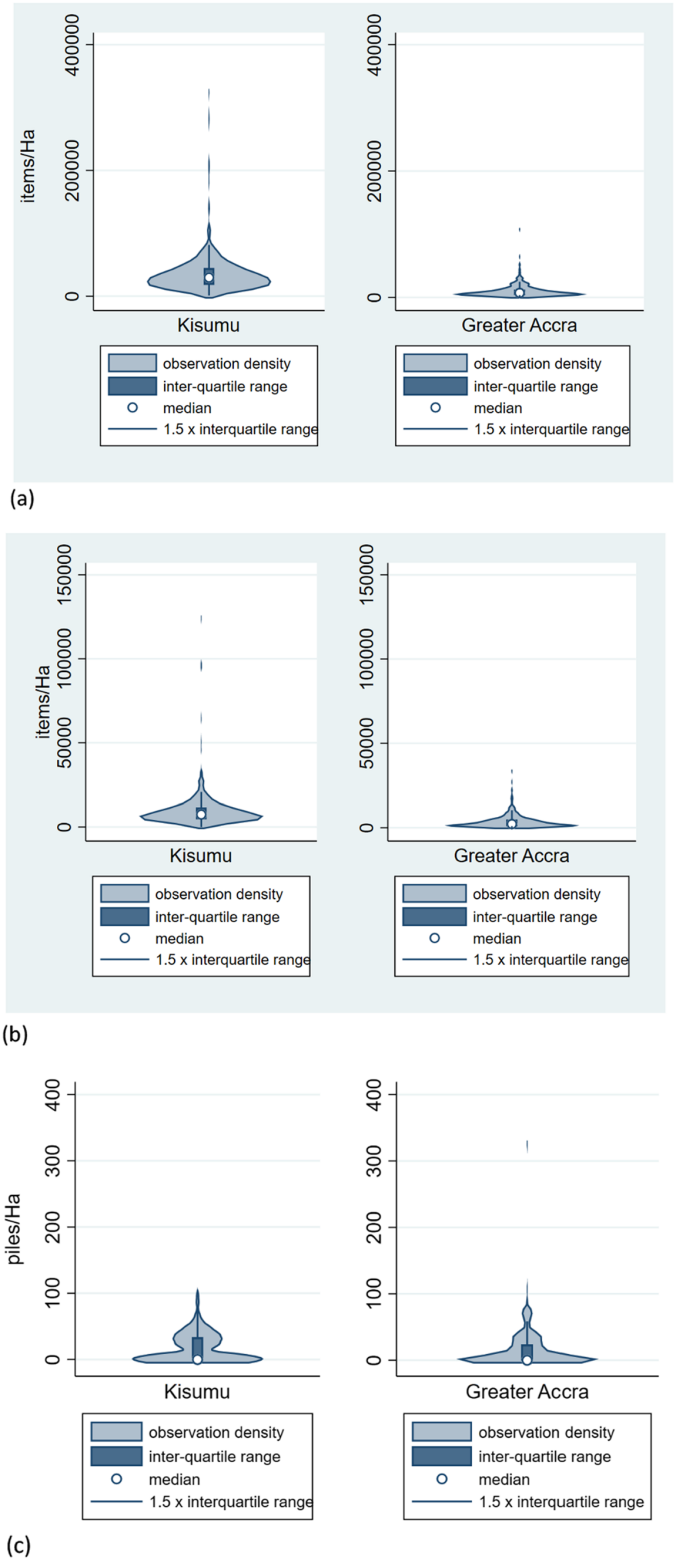
Violin plots for Greater Accra and Kisumu, showing: **a** scattered waste item density **b** density of scattered plastic waste items; **c** waste burning pile density

In both cities, mismanaged waste indicators had positively skewed distributions (Fig. 1). In Kisumu, there was a median of nearly 30,000 scattered waste items per Ha (Fig. 1a), of which 28.5% constituted plastic items (Fig. 1b). Greater Accra had approximately a third of the scattered waste density compared with Kisumu, with a higher proportion of plastic items (36%; Fig. 1b). The density of large waste piles and waste burning sites was similar across both cities, with waste burning evident along a minority of transects.

**Fig. 2:**
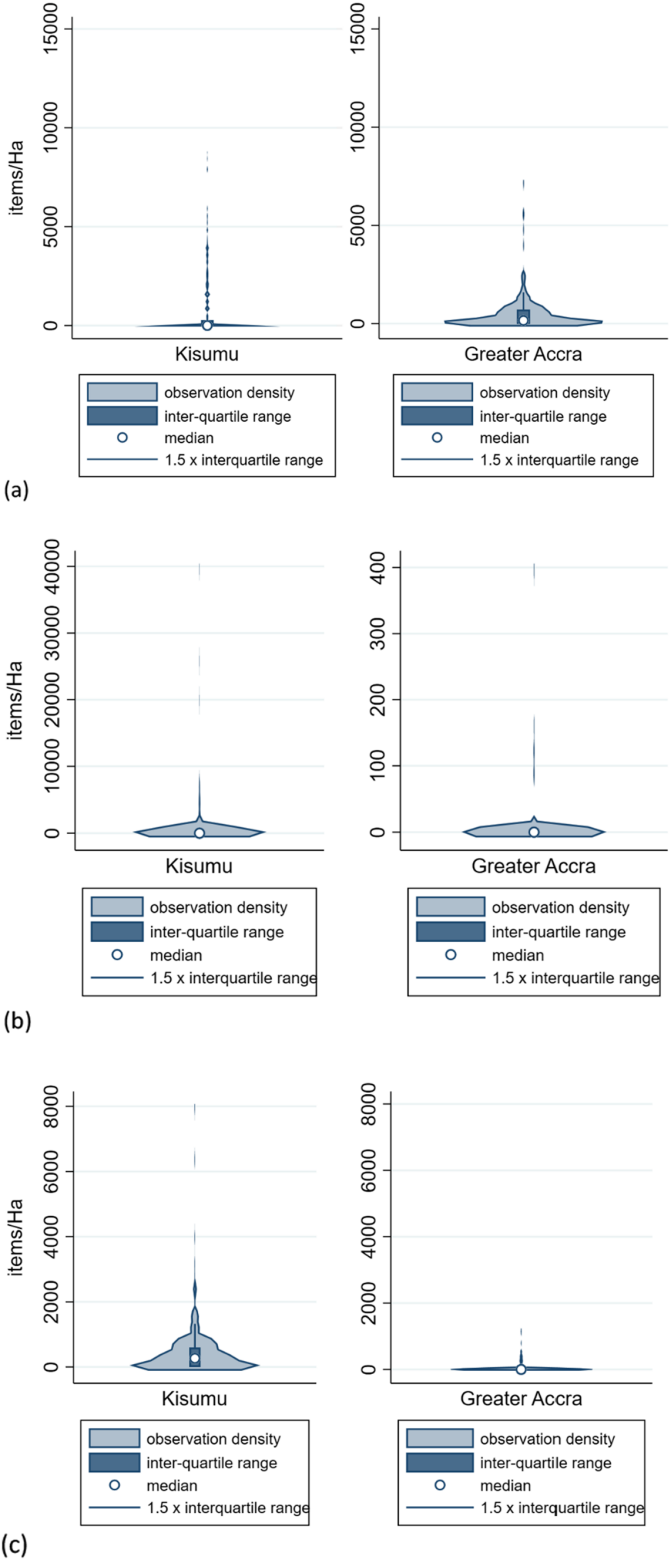
Violin plots for Greater Accra and Kisumu, showing scattered waste densities for **a** water packaging; **b** single-use disposable diapers; **c** Personal Protective Equipment

In Kisumu, median density of PPE was 263 items per Ha, with single-use nappies and discarded water packaging (Fig. 2) present as scattered waste in a minority of transects. In Greater Accra, the density of plastic water packaging items was greater, whilst the density of PPE as scattered waste was lower than in Kisumu.

Intra-class correlations from multi-level modelling suggested that correlation within transects was low for scattered waste density (0.10, 95% confidence intervals (CI) 0.06 to 0.16), proportions of plastic scattered waste (0.09, 95% CI 0.05 to 0.14), and PPE density (0.09, 95% CI 0.05 to 0.17) in Kisumu. Within-transect correlation was higher for water packaging and nappy density (0.57, 95% CI 0.52 to 0.63 and 0.33, 95% CI 0.27 to 0.40, respectively) in Kisumu. In Greater Accra, ICCs indicated no or low within-transect correlation for the proportions of plastic items in scattered waste, but moderate correlation for scattered waste, PPE, and water packaging densities (0.50, 95% CI 0.38 to 0.62; 0.47, 95% CI 0.30–0.64; and 0.55, 95% CI 0.41–0.68, respectively). Nappy density showed high within-transect correlation (0.79, 95% CI 0.70–0.85).

### Inter-observer agreement for indicators of mismanaged waste

**Fig. 3:**
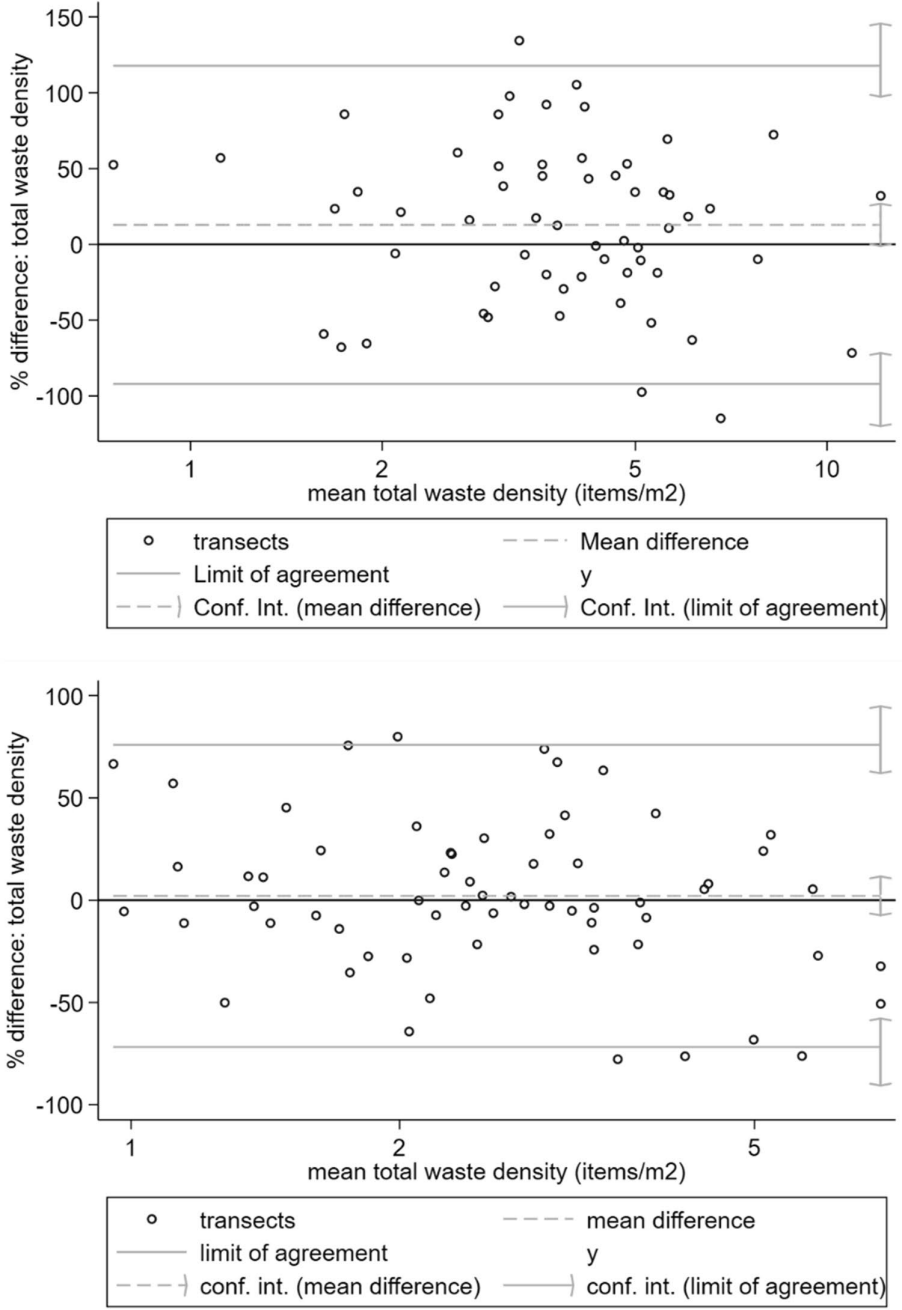

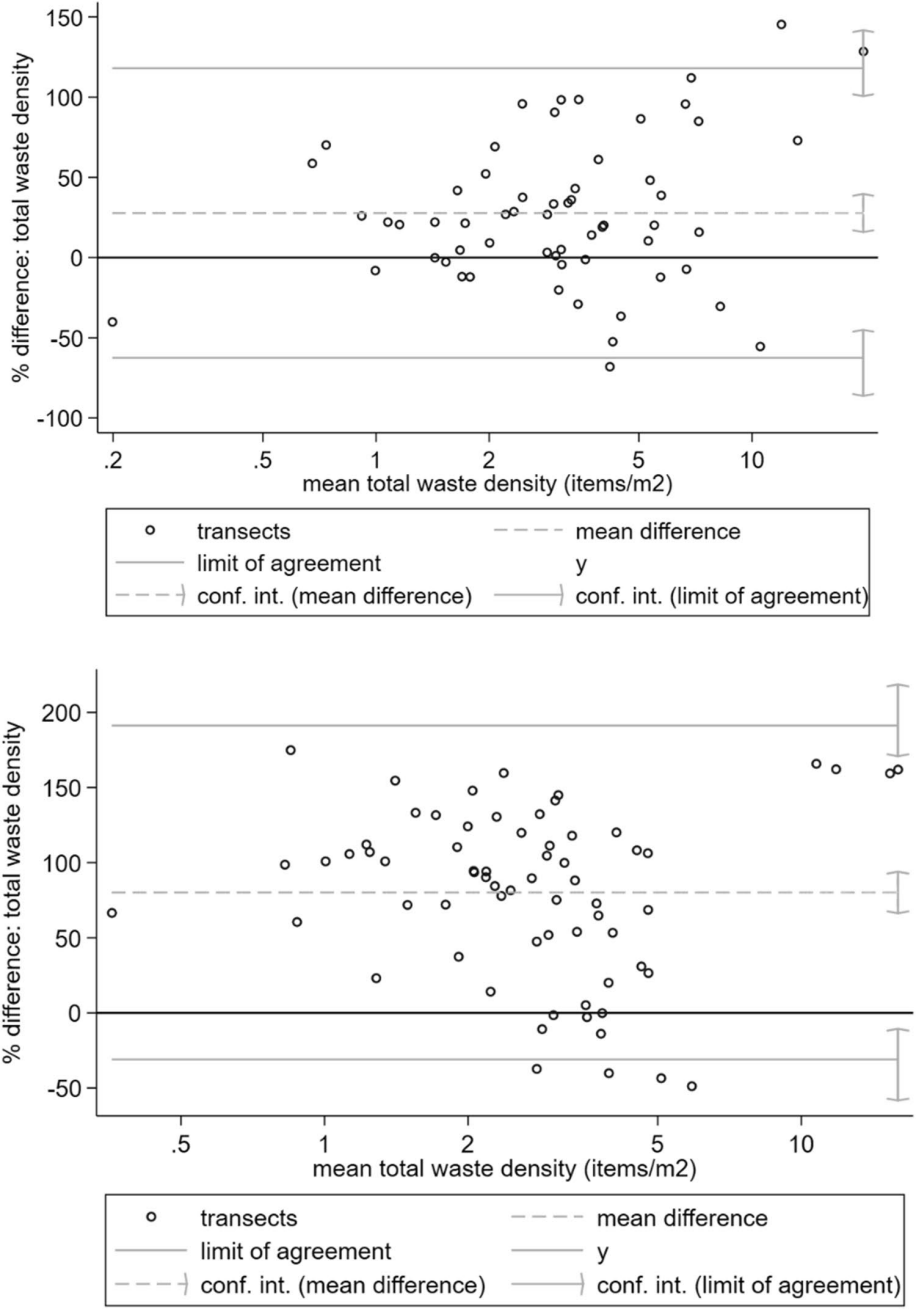
Bland and Altman limits of agreement plots for scattered waste density recorded by four pairs of transect surveyors in Kisumu, Kenya (thick solid line shows perfect agreement)

Figure 3 shows Bland and Altman plots for scattered waste densities, as recorded by the four observer pairs in Kisumu. Supplemental Figures S1 to S6 contain equivalent plots for the six observer pairs in Greater Accra. In all cases, the limits of agreement were much wider than the 20% limits proposed for beach waste surveys (Sheavly 2007). Significant bias (a mean percentage difference significantly different from zero) was apparent for two observer pairs in Kisumu and two observer pairs in Greater Accra.

**Fig. 4:**
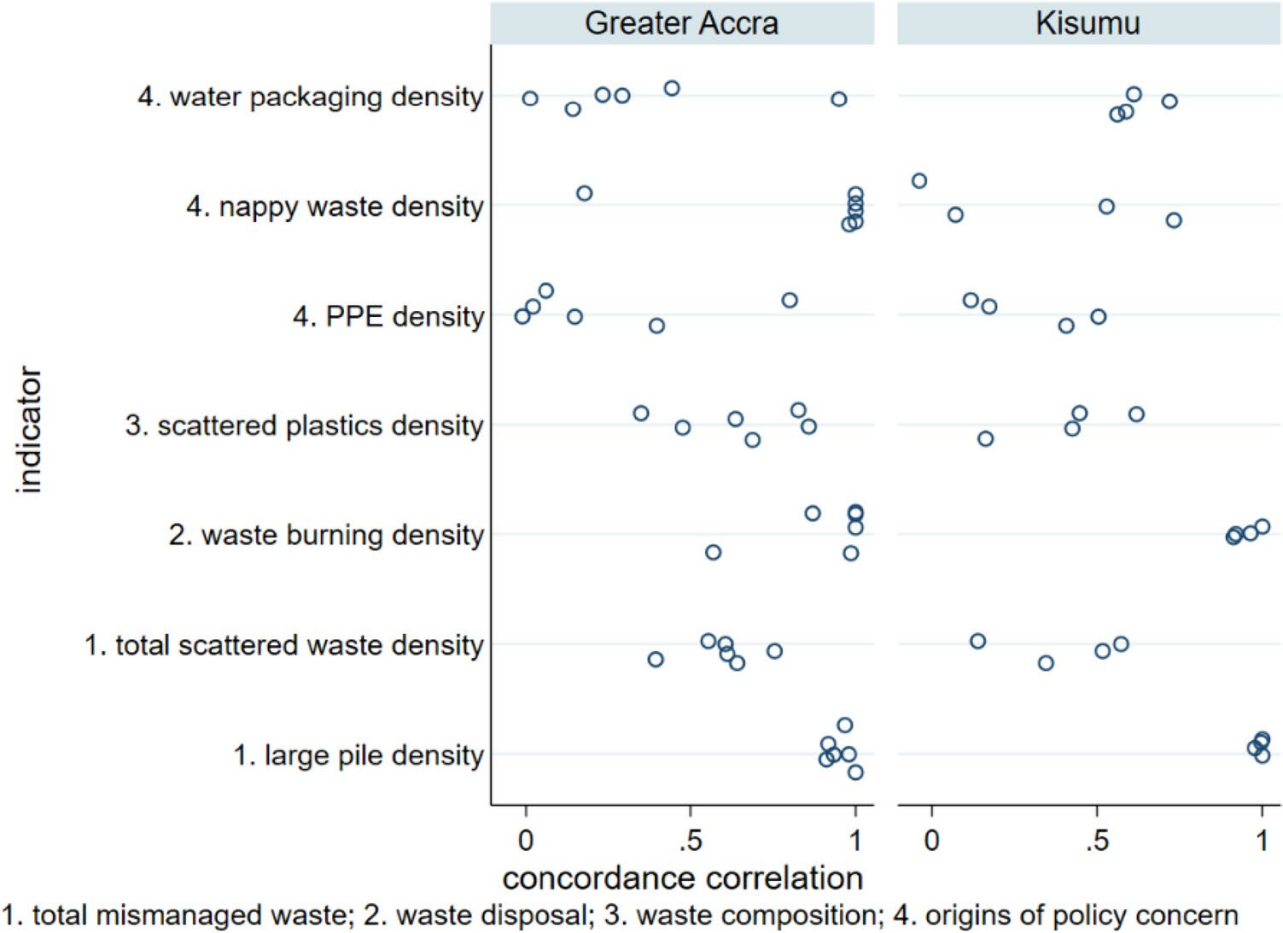
Lin’s concordance correlation coefficient for seven mismanaged waste indicators, as recorded by four observer pairs in Kisumu and six observer pairs in Greater Accra

Figure 4 shows Lin’s concordance correlation coefficient, a measure of inter-observer agreement where a value of one indicates complete agreement and zero indicates no agreement, for four surveyor pairs in Kisumu and six surveyor pairs in Greater Accra. Inter-observer agreement across the two cities showed similar patterns: it was strongest for large mismanaged waste features (large waste pile density and burnt waste pile density), somewhat weaker for total scattered waste density and scattered plastics density, and generally weakest for observations of specific waste items of policy concern (discarded PPE; nappies; and water packaging).

**Fig. 5:**
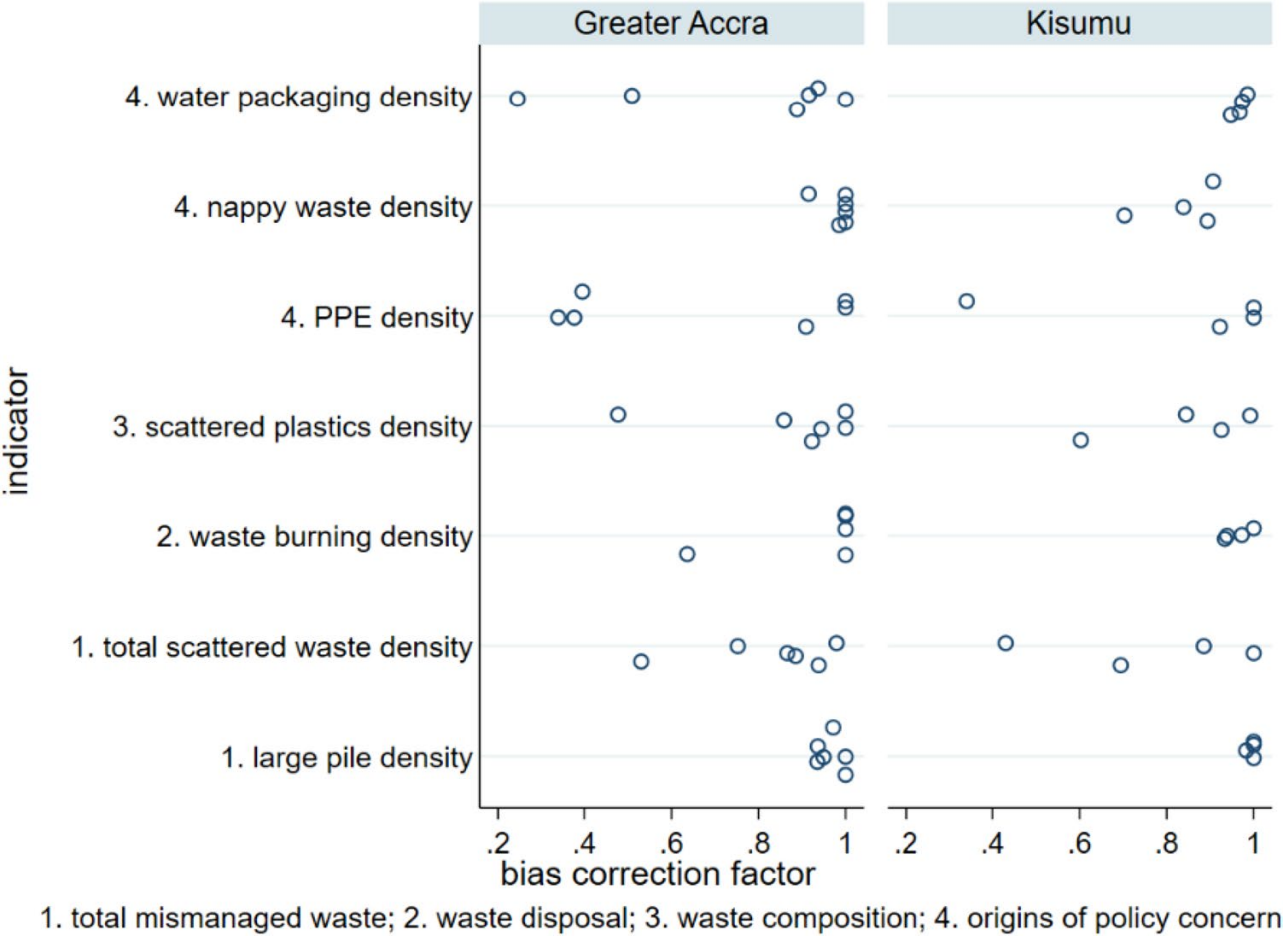
Bias correction coefficients for seven mismanaged waste indicators in slum areas, as recorded by four observer pairs in Kisumu and six observer pairs in Greater Accra

**Fig. 6:**
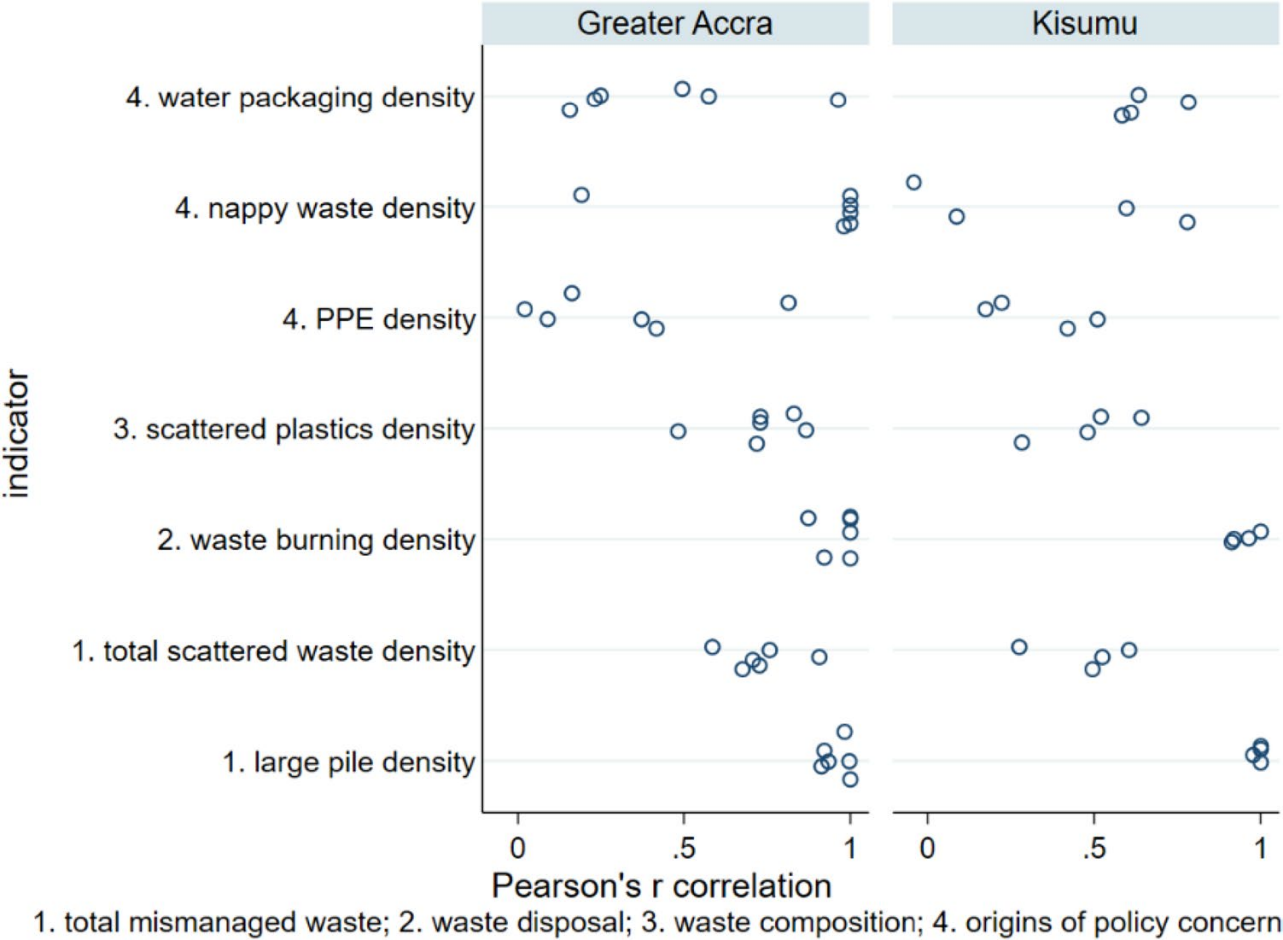
Pearson’s correlation coefficients for seven mismanaged waste indicators in slum areas, as recorded by four observer pairs in Kisumu and six observer pairs in Greater Accra

Concordance correlation coefficients are the product of the bias correction coefficient, measuring bias (Fig. 5), and Pearson’s r, a measure of precision (Fig. 6). A bias correction coefficient of one indicates no systematic under- or over-estimation of environmental waste by one observer relative to another, with lower values indicating the extent of adjustment needed to correct different observers’ waste density estimates. Generally, most bias correction coefficients were close to one, though some observer pairs showed significant under/over-estimation of total waste, scattered plastics, discarded PPE and water packaging. Observer pairs with low agreement on one indicator typically had low agreement on other indicators.

Pearson’s correlation coefficient (Fig. 6) indicated very strong correlation of burnt waste pile and large waste pile observations between surveyor pairs, moderate correlation of total scattered waste and scattered plastics density estimates and, for some observer pairs, weak or no correlation for estimates of water packaging, PPE and nappy waste density.

Some individual surveyors may have struggled to accurately record waste in the field, since the same observer pairs generally showed consistent bias and low concordance across multiple waste indicators. The use of pacing to select transect locations for spot counts of scattered waste meant observers were not always observing waste at precisely the same location along a transect, for example because of the challenging slum environment or stride length differences between individuals. Low inter-observer agreement in some cases thus likely also reflected spatial variation in scattered waste densities along transects. Counting waste along the entire transect rather than at spot locations would overcome this issue, but be more labour-intensive and time consuming. Alternatively, observers with similar stride lengths could be paired. In Kisumu, there was generally higher concordance for water packaging observations (Fig. 3), the indicator which also showed the greatest within-transect correlation in multi-level modelling. This suggests inter-observer differences partially reflect spatial variation in scattered waste within transects.

### Inter-observer disagreement impacts on cross-city comparison of mismanaged waste

When robust regression was used to test for significant differences in waste indicators between Kisumu and Greater Accra, randomly selecting data from one observer within each pair and repeating the analysis for the second observer set (Table 1), these comparisons were generally not sensitive to the choice of observers. For both random observer sets, scattered waste density and density of scattered plastics were significantly greater in Kisumu, as was PPE scattered waste density. Density of discarded disposable nappies was marginally significantly higher in Kisumu for one observer set, and higher but not significantly so for the other observer set. There were no significant differences between cities in other waste indicators.

**Table 1:** Robust regression coefficients, comparing mismanaged waste indicators between Kisumu and Greater Accra (positive coefficients indicate higher indicator values in Kisumu)

	Observer set 1		Observer set 2	
Mismanaged waste domain and indicators	Coefficient	P value	Coefficient	P value
*Domain: Total mismanaged waste*				
Scattered waste density (items/Ha)	21,192 (13,400 to 28,985)	< 0.001	25,316 (19,790 to 30,842)	< 0.001
*Domain: waste composition*				
Density of plastic scattered waste items (items/Ha)	6093.6 (3884.6 to 8302.6)	0.64	4074.2 (2773.5 to 5375.0)	< 0.001
*Domain: waste disposal model*				
Burnt waste site density (piles/Ha)	3.79 (-3.8 to 11.39)	0.32	1.53 (-7.27 to 10.33	0.35
Large waste dumps (piles/Ha)	4.09 (-0.96 to 9.14)	0.11	4.19 (-0.72 to 9.10)	0.09
*Domain: origins of policy concern*				
PPE density (items/Ha)	368.9 (279.3 to 458.5)	< 0.001	275.5 (212.1 to 339.1)	< 0.001
Nappy density (items/Ha)	288.5 (31.9 to 545.0)	0.03	325.8 (-31.1 to 682.7)	0.07
Water packaging density (bottles or sachet sleeves/Ha)	226.8 (-282.1 to 735.8)	0.37	92.1 (-279.9 to 464.1)	0.62

The results in Table 1 suggest that the magnitude of inter-city variation in waste counts exceeded inter-observer bias in transect surveys, enabling identification of city-level differences in waste indicators. It also suggests that transect surveys are sufficiently robust for targeting waste management actions or environmental clean-up resources at city level, for example, via the UNEP plastic hot-spotting approach (Boucher et al. 2020).

### Implications of inter-observer agreement for recording environmental waste

This study is the first examination of inter-observer reliability for surveys of mismanaged waste in the environment. Most urban or beach environmental waste transect surveys do not assess inter-observer agreement (van Gool et al. 2021; Araújo et al. 2018; de Ramos et al. 2021; Kalnasa et al. 2019; Okuku et al. 2021). The current study finds very high agreement between observers in identifying waste burning sites and large waste sites (Fig. 2). This finding is encouraging for studies that could use such observations as “ground truth” or calibration data for satellite image classification (Georganos et al. 2021) and for those estimating waste burning emissions from municipal waste burning surveys (Das et al. 2018; Nagpure et al. 2015). There was more moderate inter-observer agreement of total and plastic scattered waste per transect, with lower inter-observer agreement for waste of specific origins (PPE, water packaging, and nappies), which occurs in lower densities at the locations investigated in the present study. Bland and Altman limits of agreement for percentage difference in this study’s scattered waste measurements are much wider than the 20% acceptable limit proposed for beach litter surveys (Sheavly 2007), reflecting challenging field conditions in slums, including interruptions from by-standers and difficulty of navigation. There is also evidence of inter-observer bias, i.e. under- or over-reporting of waste indicators, by a minority of surveyors (Figs. 3 and 5). Despite these biases, statistical comparisons between waste indicators for Greater Accra and Kisumu were, however, not sensitive to the choice of observer data set used.

Very few waste transect studies document efforts to assess or control for inter-observer reliability and the few that do either try to maintain the same survey personnel (Willis et al. 2017) or use a mean difference of 20% in waste counts as a quality control threshold between observers (Sheavly 2007). Since such measures do not enable observer bias (systematic under- or over-reporting of waste) to be distinguished from precision, use of Bland and Altman limits of agreement analysis is therefore recommended for pre-survey quality control and observer standardisation. With several mobile phone applications now available for environmental waste surveys (Zettler et al. 2017), particularly via citizen science, there would further be scope to build support for inter-observer reliability assessment into such software. Some studies examine spatial or temporal differences in standing waste loads between sites to infer sources or rates of mismanaged waste entry into the environment or its subsequent transport (Olivelli et al. 2020; van Gool et al. 2021; Mugilarasan et al. 2021). Some instances were identified of apparent under-reporting of waste counts by observers (Fig. 3). In household survey analysis, techniques have been developed that use paradata (i.e. data on survey implementation processes, such as start- and end-times of transect observations or observer identity) to assess measurement error (Yan and Olson 2013; Da Silva and Skinner 2021). Therefore, where initial inter-observer standardisation or quality control is not possible because of limited resources, future studies are recommended to consider or control for observer identity using paradata when analysing spatial or temporal variation in standing waste loads or composition.

### Implications for waste management in urban sub-saharan Africa

For beach surveys, a clean coast index has been developed, which classifies beaches as “dirty” where plastic waste item densities exceed 0.5 parts/m^2^ and “extremely dirty” where densities exceed 1 part/m^2^ (Alkalay et al. 2007). Applying this index to the present study’s urban transects, 33.1% of Kisumu’s transects were extremely dirty and 38.6% dirty, whilst 6.7% of Greater Accra’s transects were extremely dirty and 15.5% dirty. Median scattered waste densities are approximately three times greater in Kisumu with higher densities of scattered plastics, reflecting lower waste service coverage in Kisumu relative to Greater Accra. Alongside the contrast between Accra as a capital and Kisumu as a provincial city, the two countries have contrasting plastic management policies. Kenya has sought to limit plastic production and consumption since 2005, though often with opposition from a powerful plastics manufacturing lobby (Behuria 2021). Packaged water consumption in Kenya remains low, whilst Ghana has tolerated a packaged (sachet or bottled) drinking water industry that constituted the main drinking water source for 58% of urban Ghanaian households in 2019 (Ghana Statistical Services and ICF 2020a). The proportion of plastics in scattered waste is lower in Kisumu than Greater Accra, which could in part reflect these contrasting national policies. Specifically, water packaging constituted a mean 5.0% of scattered waste in off-grid Greater Accra, but 2.5% of scattered waste in Kisumu, reflecting widespread water sachet consumption in urban Ghana.

In Kisumu in particular, there was further evidence that uptake of WASH-related consumer products was creating waste management challenges, reflecting lifestyles among communities lacking services. There were locally high densities of single-use nappies in Kisumu’s environment, with 39,789 nappies/Ha in some transects. Anecdotal evidence suggested that, in recent years, Kenyan single-use nappy prices have fallen sufficiently to enable their purchase by lower income households. Elsewhere, disposable nappy use has been observed in an Indonesian slum and significantly associated with unhygienic child faeces disposal (Agestika et al. 2021). Disposable nappy use in low-income neighbourhoods has also been reported as an emerging concern for municipal waste services in Bulawayo, Zimbabwe (Mbiba 2014). This highlights the emergence in multiple cities of a nexus between solid waste management and WASH-related lifestyle changes. Classification of both child faeces disposal as solid waste (Bain and Luyendijk 2015) and consumption of packaged water (Stoler 2012) has proven difficult when designing international monitoring indicators relating to Sustainable Development Goal 6 (clean water and sanitation for all). Given evidence here that both are locally significant contributors to urban mismanaged solid waste standing loads in Greater Accra and Kisumu, there is thus a case for greater dialogue and integration between the WASH and solid waste management sectors for both policy and service delivery planning.

The current study confirms evidence from multiple countries and desk-based global estimates (Chowdhury et al. 2021) of increased hygiene-related waste entering the environment due to the COVID-19 pandemic. A street survey of coastal Kenyan towns found that pandemic-related waste (including sanitiser bottles, hand wipes, and PPE) constituted < 100 items/Ha, at levels of up to 900 items/Ha (Okuku et al. 2021), whilst in urban South Africa, hygiene-related waste constituted 5.3% of scattered waste items during lockdown (Ryan et al. 2020). Surveyors in this study did not count hygiene-related items such as wipes or sanitiser bottles, but found higher median PPE waste in Kisumu of 265 items/Ha, at levels of up to 7958 items/Ha per transect (Fig. 2), with lower discarded PPE densities in Greater Accra of up to 1194 items/Ha (Table 1). Locally, per transect, PPE constituted up to 13% and 56% of scattered waste in Greater Accra and Kisumu, respectively, confirming a locally substantial increase in mismanaged hygiene-related waste because of the pandemic.

### Limitations and generalisability

This study was conducted in circumstances that constrained some aspects of the observations. In evaluating inter-observer agreement, for their personal safety, surveyors visited field sites in pairs rather than separately. Although field supervisors and surveyors were instructed to avoid collusion between team members, this could still potentially have occurred, inflating inter-observer reliability estimates. Despite careful community sensitisation, the presence of survey teams could also have affected community behaviours, triggering waste clean-up, thereby leading to under-estimation of standing waste loads. There were also some protocol deviations in Greater Accra for staffing reasons that could have influenced inter-observer agreement estimates. In particular, two EAs were surveyed by only one staff member and some staff moved between pairs in some instances. To protect field staff from handling waste that could be contaminated with COVID-19 during the pandemic, the study protocol used remote observation of environmental waste (Cheshire et al. 2009), rather than picking up and weighing waste items via an accumulation study design (Opfer et al. 2012). In Greater Accra particularly, it is common for waste to be wrapped in plastics before disposal; hence, survey teams were unable to unwrap such waste items. Indicators may therefore over-estimate the proportion of plastics in scattered waste, but under-estimate discarded nappies, water packaging, or other forms of waste where these were wrapped in plastics.

Off-grid urban areas are challenging field environments, where personal security threats and groups of by-standers may distract survey teams, whilst densely packed housing restricts observer visibility and makes navigation challenging (Yentumi et al. 2019). Thus, inter-observer reliability is likely to be lower in the current study than in beach surveys or in formally planned urban settlements. Since survey teams observed but did not collect or weigh waste to minimise COVID-19 risks, reliability estimates are not generalisable to surveys that collect and weigh waste items. All members of survey teams had received degree level education, so whilst this study’s findings are likely relevant to research and professionally organised transect surveys, inter-observer agreement may differ for surveys conducted by others with different educational backgrounds or experience.

## Conclusion

Through future studies, there would be scope to assess how far the use of quality control measures (e.g. initial inter-observer standardisation exercises; standardisation of stride length) increased reliability of waste indicator data. This study drew on a proposed acceptable level of inter-observer agreement from beach litter surveys (Sheavly 2007). In future, through assessment of inter-observer agreement’s impacts on spatio-temporal waste flux analyses of environmental waste surveys, research could also explore what constitutes acceptable inter-observer agreement based on such sensitivity analysis. Such sensitivity analysis could also be used to assess inter-observer agreement where all waste is counted along a transect. Since high-resolution gridded population map layers are now available for urban areas (Leyk et al. 2019), future studies could calculate and analyse waste densities per capita from transect surveys, as well as per unit area. The present study provides evidence of waste entering the urban environment from products such as disposable nappies and bottled or bagged water. Given this apparent nexus between WASH and mismanaged waste, greater consideration should be given to integrating solid waste management within WASH service delivery and monitoring.

This study involving observer pairs recording environmental waste in slums finds that inter-observer agreement levels for scattered waste indicators are generally much wider than the 20% allowable difference between observers recommended for beach litter surveys. In both Kisumu and Greater Accra, there was excellent inter-observer reliability for densities of burnt waste and large waste piles (concordance correlation coefficient > 0.9 for all but one observer pair). However, inter-observer reliability varied from weak to excellent for scattered waste density (concordance correlation coefficients 0.39 to 0.98), and densities of specific waste items such as disposable nappies and discarded PPE (concordance correlation coefficients 0.28 to 0.99). This likely reflects challenging field conditions for survey teams in slums. Some observers also systematically under- or over-estimate scattered waste. Inter-observer agreement concerning larger features such as waste pile and waste burning site density is, however, much greater. Despite issues such as observer bias when recording scattered waste, waste indicator comparisons between Greater Accra and Kisumu were, however, not sensitive to random selection of one observer record set over another. This indicates that beach litter survey protocols, as developed by UNEP and NOAA, can successfully be adapted to monitor mismanaged waste in urban environments, including the challenging field conditions encountered in slums. Transect survey findings can be used to target geographic hot-spots of waste for environmental clean-up or upgrading of waste management infrastructure and services at city level. Survey findings also provide evidence of application hot-spots (Boucher et al. 2020), such as plastics from bottled or sachet water.

Finally, the study provides evidence that WASH-related consumer products, namely single-use nappies, water packaging from sachets or bottles, and hygiene products, are increasingly entering the urban environment as mismanaged waste as consumption patterns change in low-income urban areas lacking waste collection services. This issue urgently requires innovative solutions and highlights the need to expand waste collection service delivery into slum areas. It also highlights the need for integrated planning of urban water, sanitation, and domestic waste collection service delivery.

## Supplementary Information

Below is the link to the electronic supplementary material.

Supplementary file1 (TIF 152 KB)

Supplementary file2 (TIF 154 KB)

Supplementary file3 (TIF 140 KB)

Supplementary file4 (TIF 144 KB)

Supplementary file5 (TIF 144 KB)

Supplementary file6 (TIF 146 KB)

Supplementary file7 (TIF 142 KB)

Supplementary file8 (TIF 140 KB)

Supplementary file9 (TIF 142 KB)

Supplementary file10 (TIF 142 KB)

Supplementary file11 (TIF 162 KB)

Supplementary file12 (TIF 162 KB)

Supplementary file13 (TIF 161 KB)

Supplementary file14 (DOCX 93 KB)

## Data Availability

Environmental transect data for this study are available from the UK Data Archive via the following link: https://dx.doi.org/10.5255/UKDA-SN-856145.

## Abbreviations

CI: Confidence Interval
COVID-19: Coronavirus Disease 2019
EA: Enumeration Area
GPS: Global Positioning Systems
GSV: Google StreetView
ICC: Intra-Class Correlation
NOAA: National Oceanographic and Atmospheric Organisation
PPE: Personal Protective Equipment
UAV: Unmanned Aerial Vehicle
UNEP: United Nations Environment Programme
WASH: Water, Sanitation, and Hygiene
