# Supplementary figures and images for: Inter-observer reliability in transect-based observations of environmental waste in greater accra and kisumu: implications for waste management
Source: Int J Environ Sci Technol (Tehran). 2024 Apr 22;21(15):9409–24. doi: 10.1007/s13762-024-05625-5 (PMC11480156; doi:10.1007/s13762-024-05625-5)

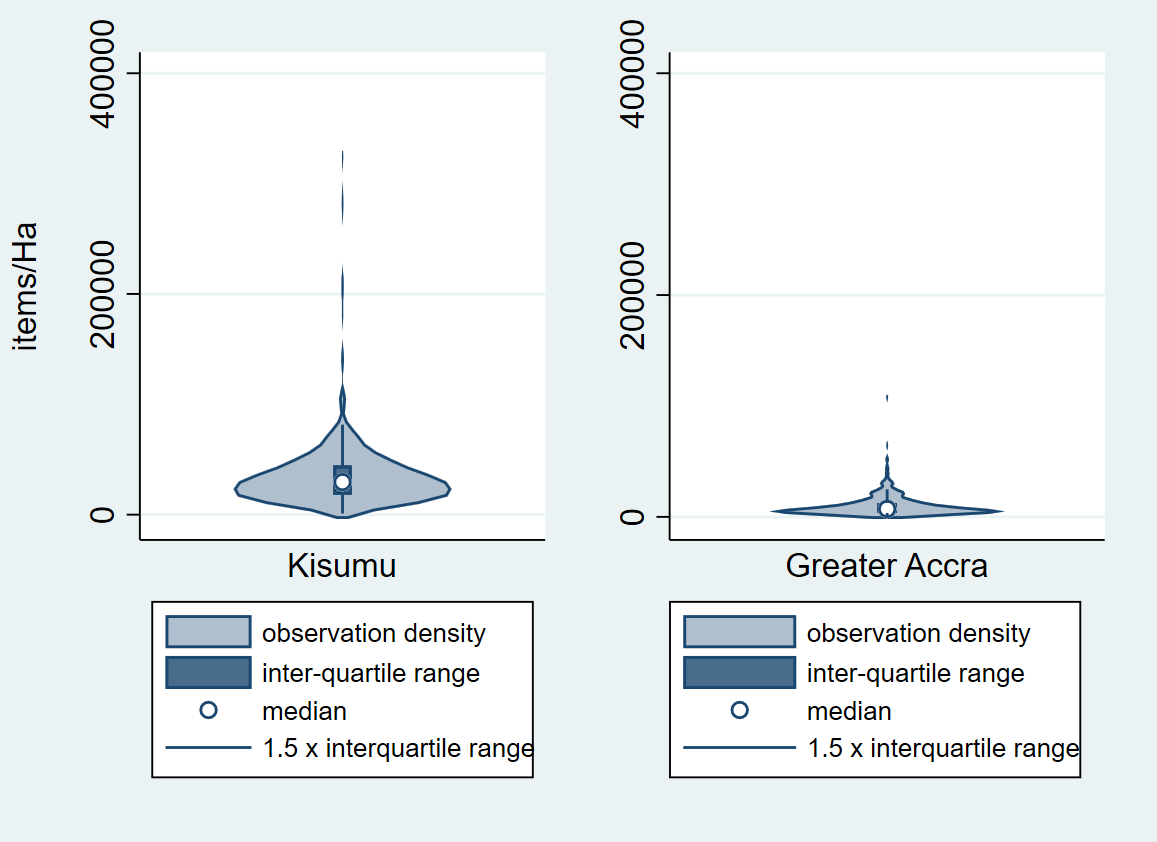

Supplement: Supplementary file 1 — Supplementary file1 (TIF 152 KB) [file 13762_2024_5625_MOESM1_ESM.tif]

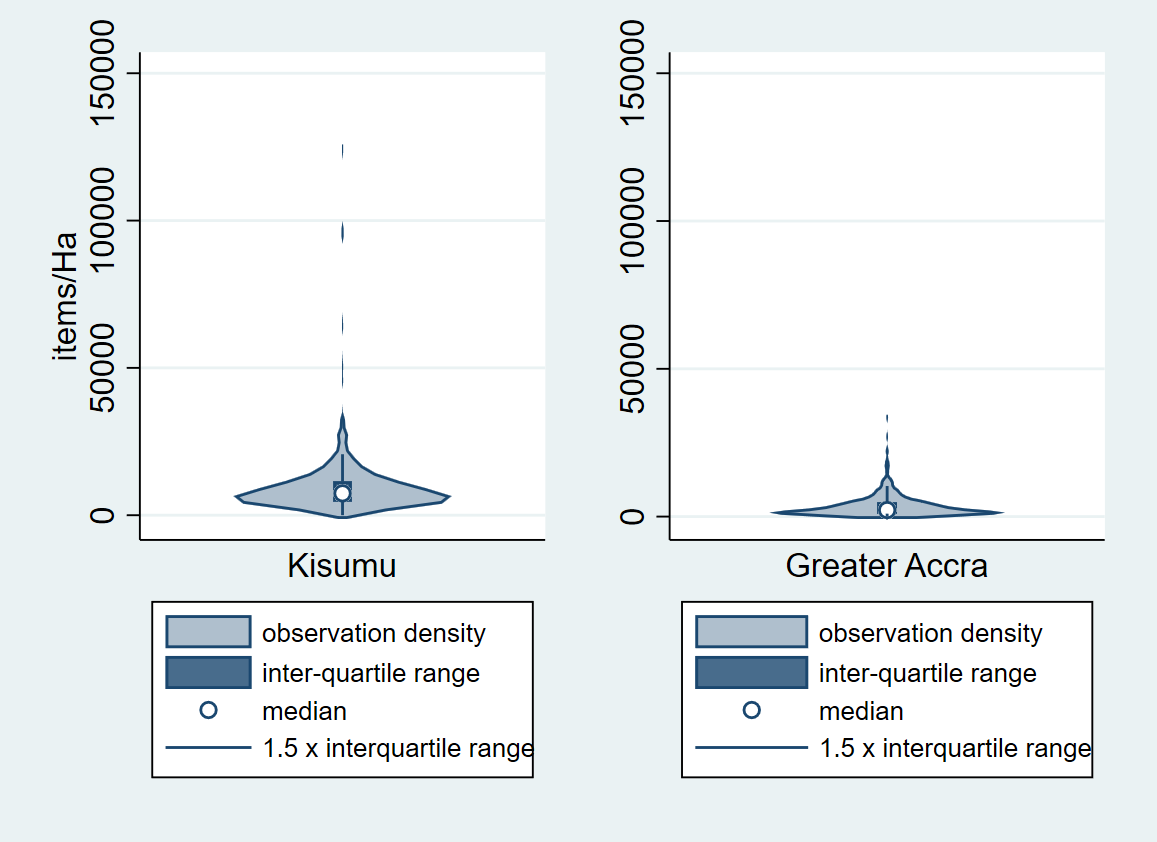

Supplement: Supplementary file 2 — Supplementary file2 (TIF 154 KB) [file 13762_2024_5625_MOESM2_ESM.tif]

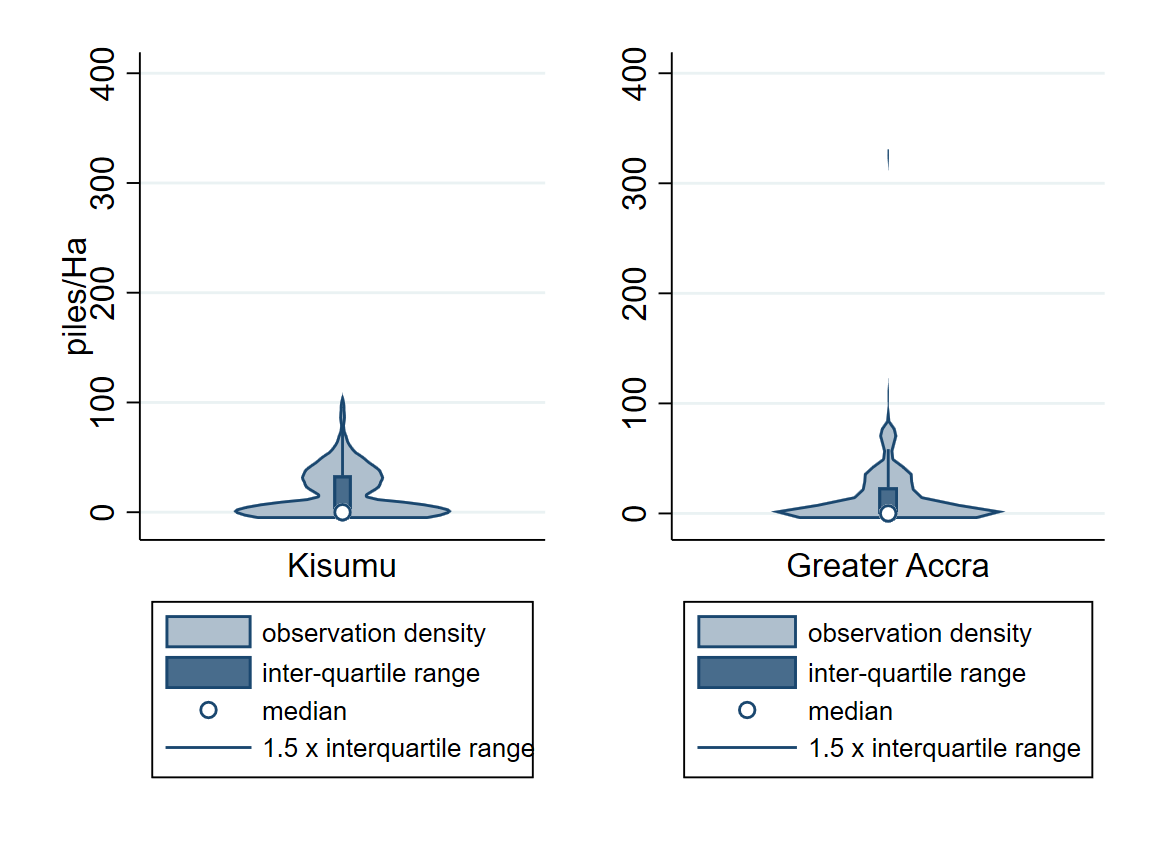

Supplement: Supplementary file 3 — Supplementary file3 (TIF 140 KB) [file 13762_2024_5625_MOESM3_ESM.tif]

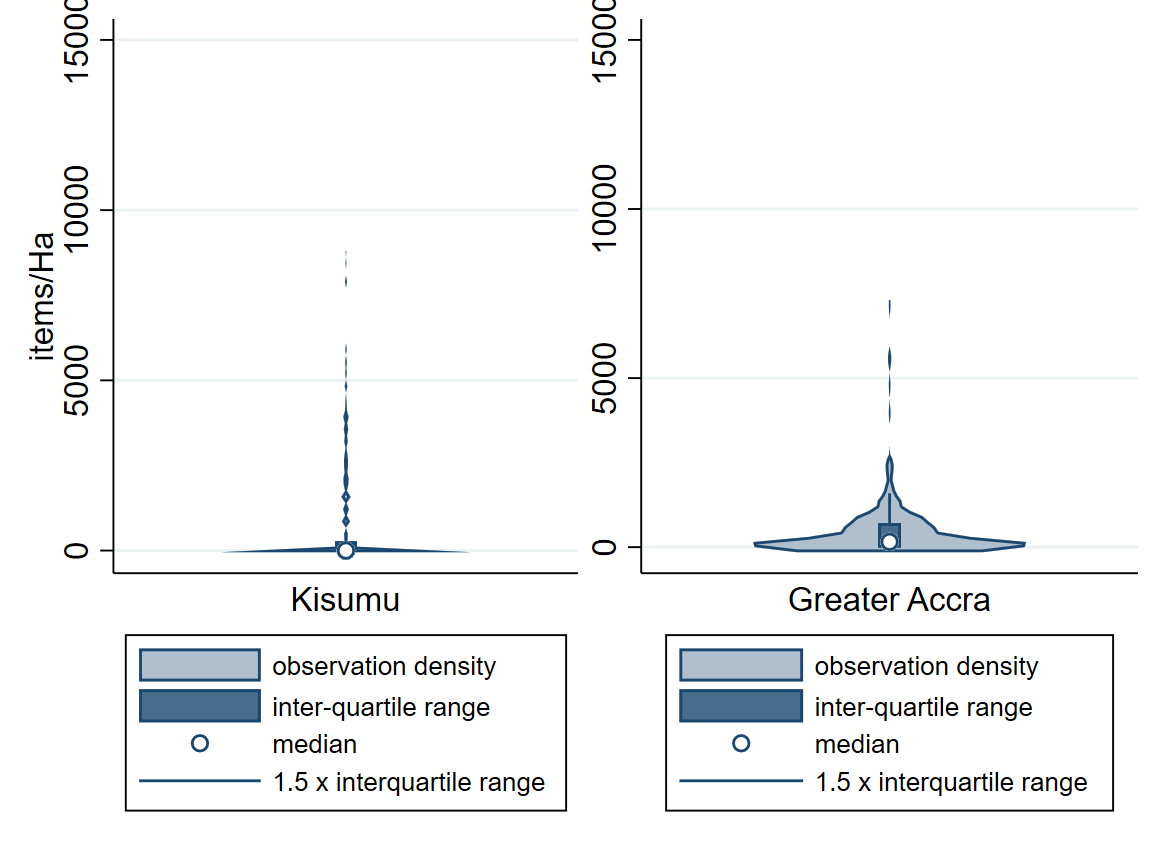

Supplement: Supplementary file 4 — Supplementary file4 (TIF 144 KB) [file 13762_2024_5625_MOESM4_ESM.tif]

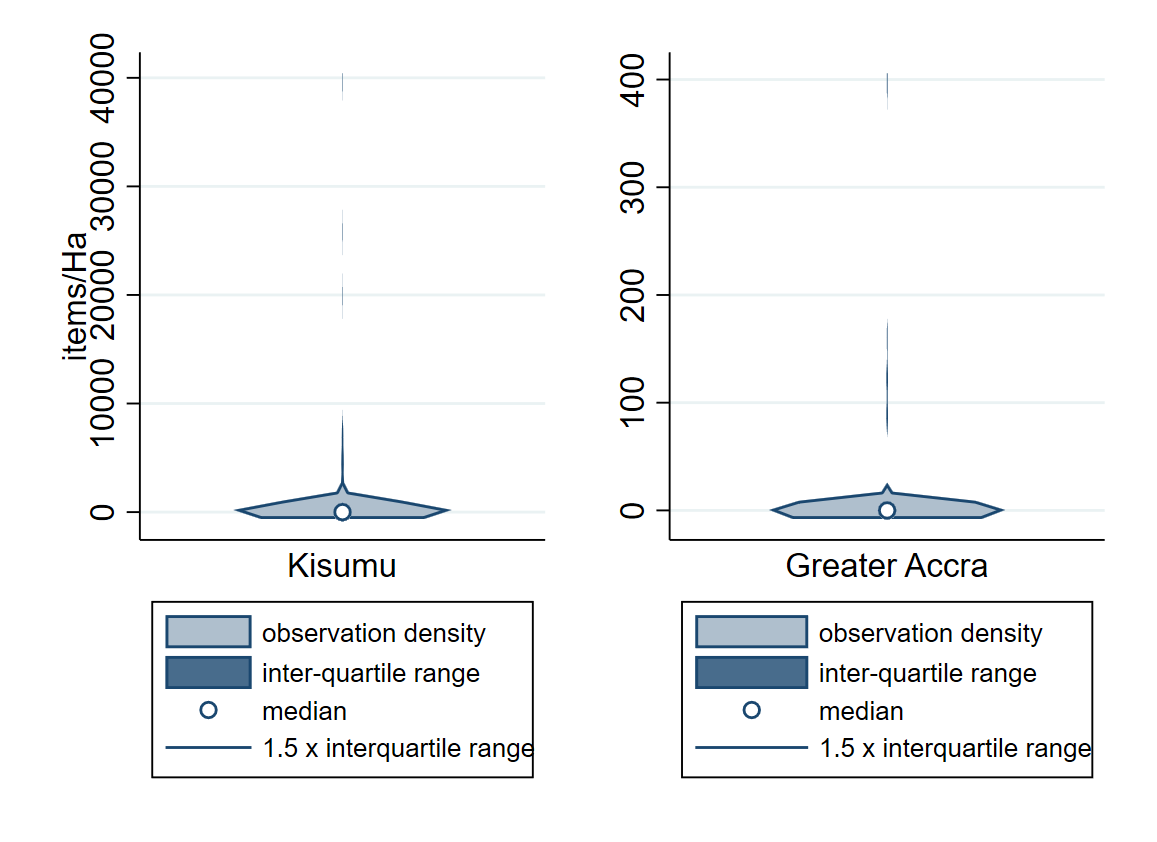

Supplement: Supplementary file 5 — Supplementary file5 (TIF 144 KB) [file 13762_2024_5625_MOESM5_ESM.tif]

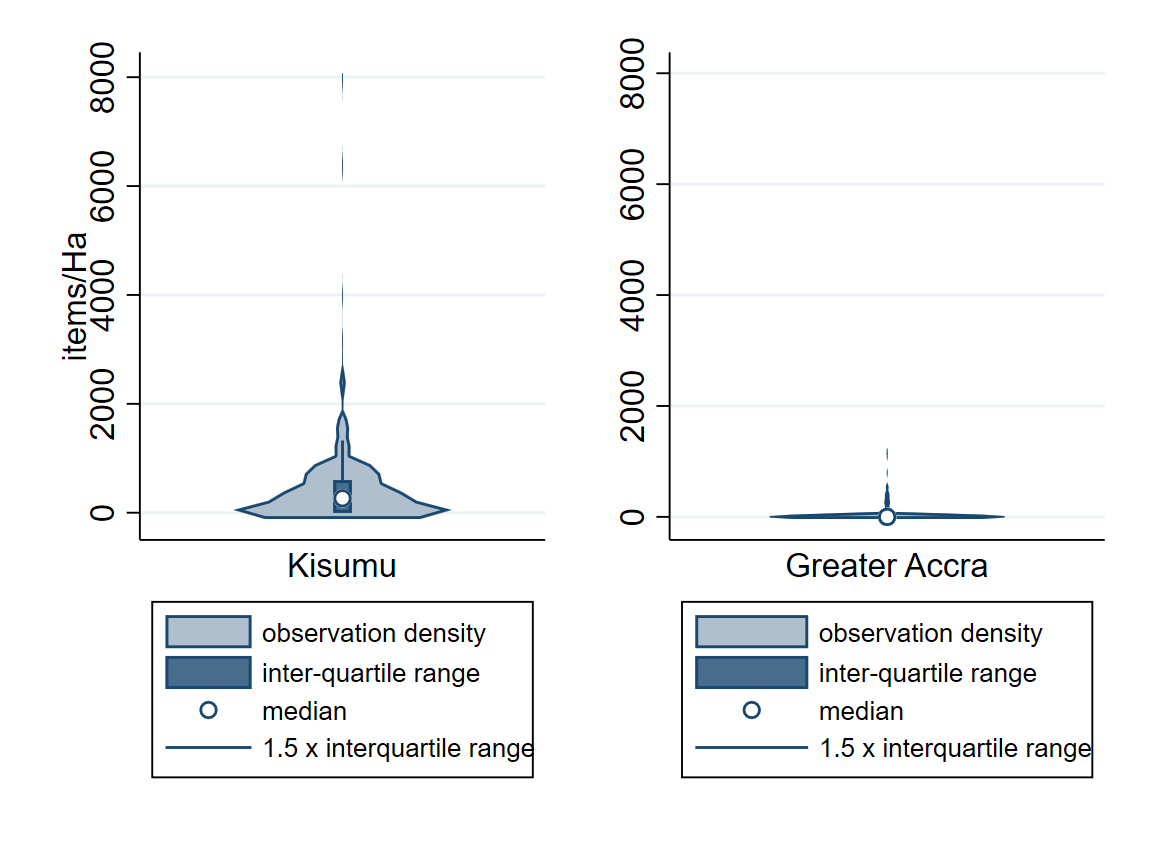

Supplement: Supplementary file 6 — Supplementary file6 (TIF 146 KB) [file 13762_2024_5625_MOESM6_ESM.tif]

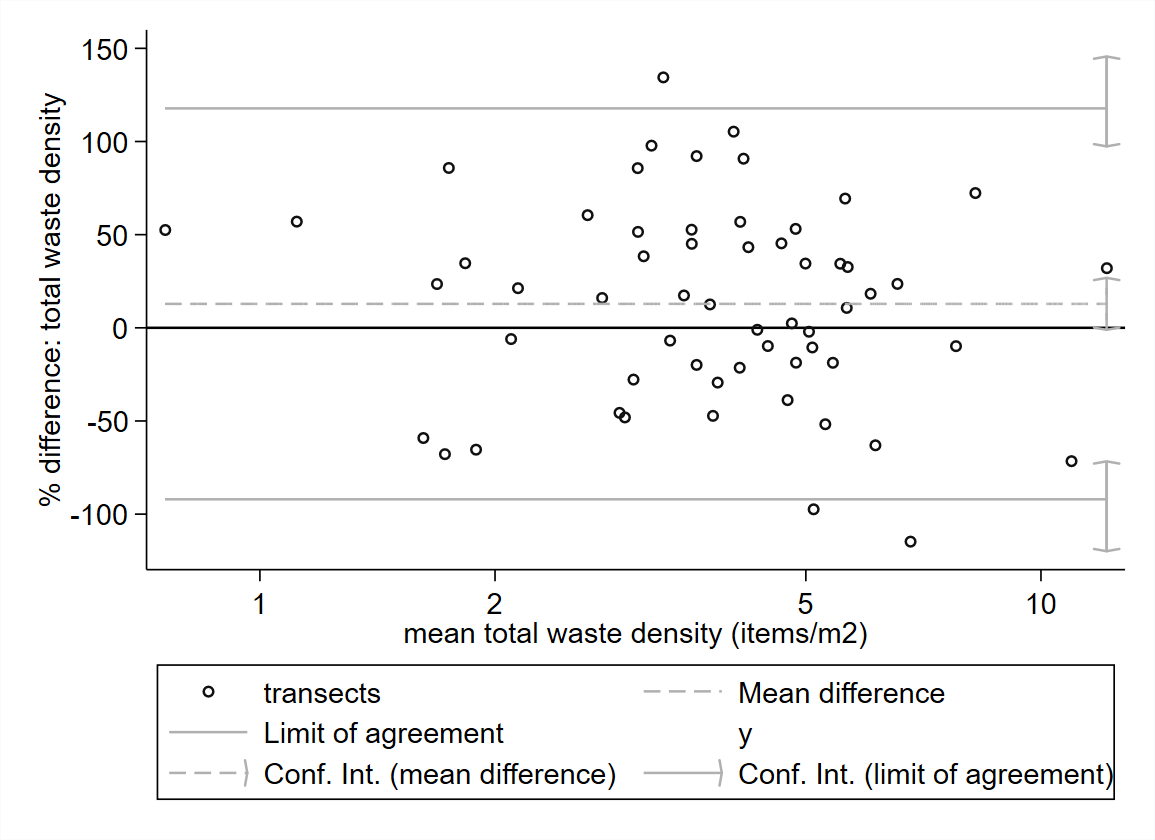

Supplement: Supplementary file 7 — Supplementary file7 (TIF 142 KB) [file 13762_2024_5625_MOESM7_ESM.tif]

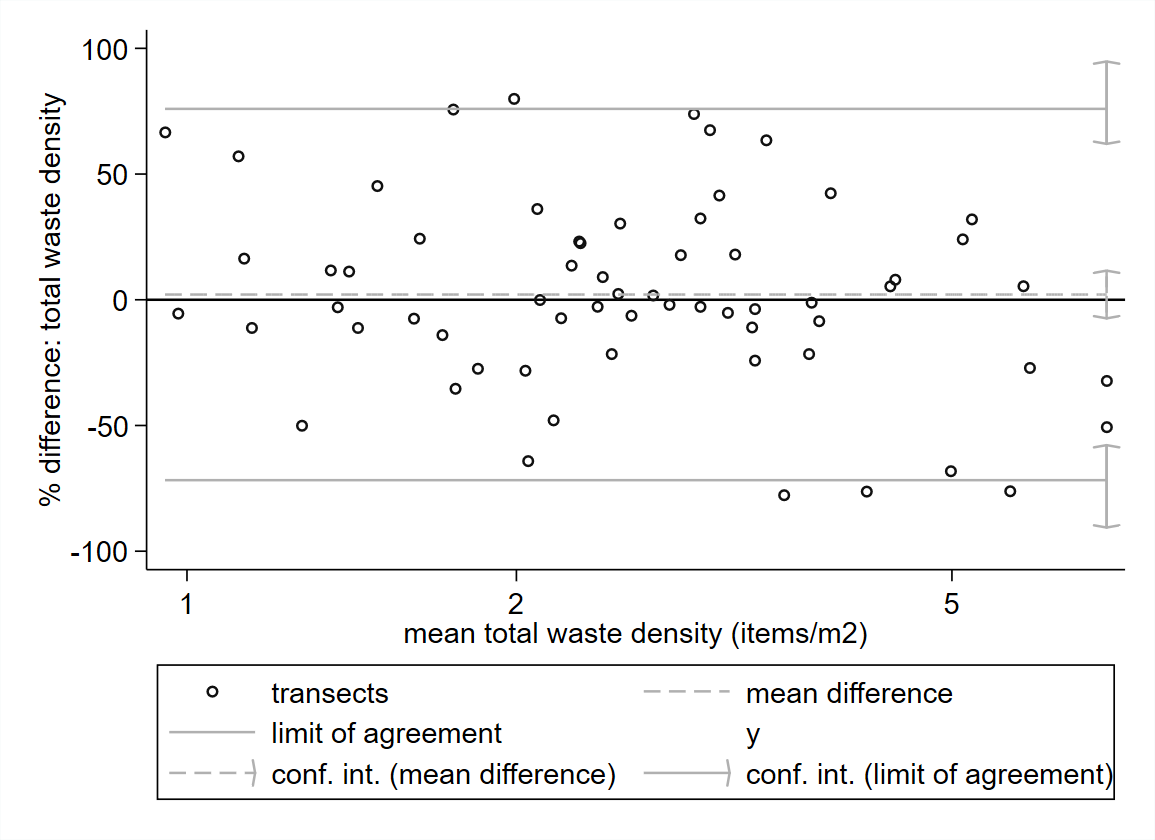

Supplement: Supplementary file 8 — Supplementary file8 (TIF 140 KB) [file 13762_2024_5625_MOESM8_ESM.tif]

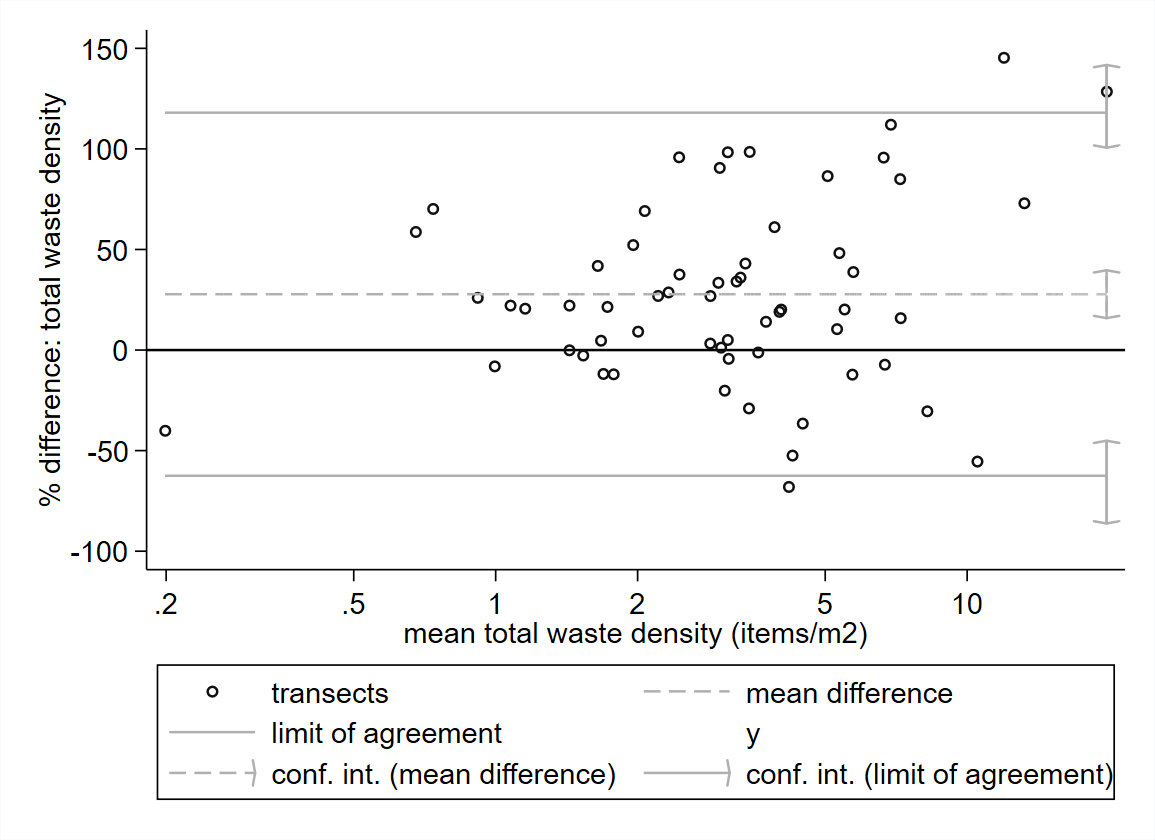

Supplement: Supplementary file 9 — Supplementary file9 (TIF 142 KB) [file 13762_2024_5625_MOESM9_ESM.tif]

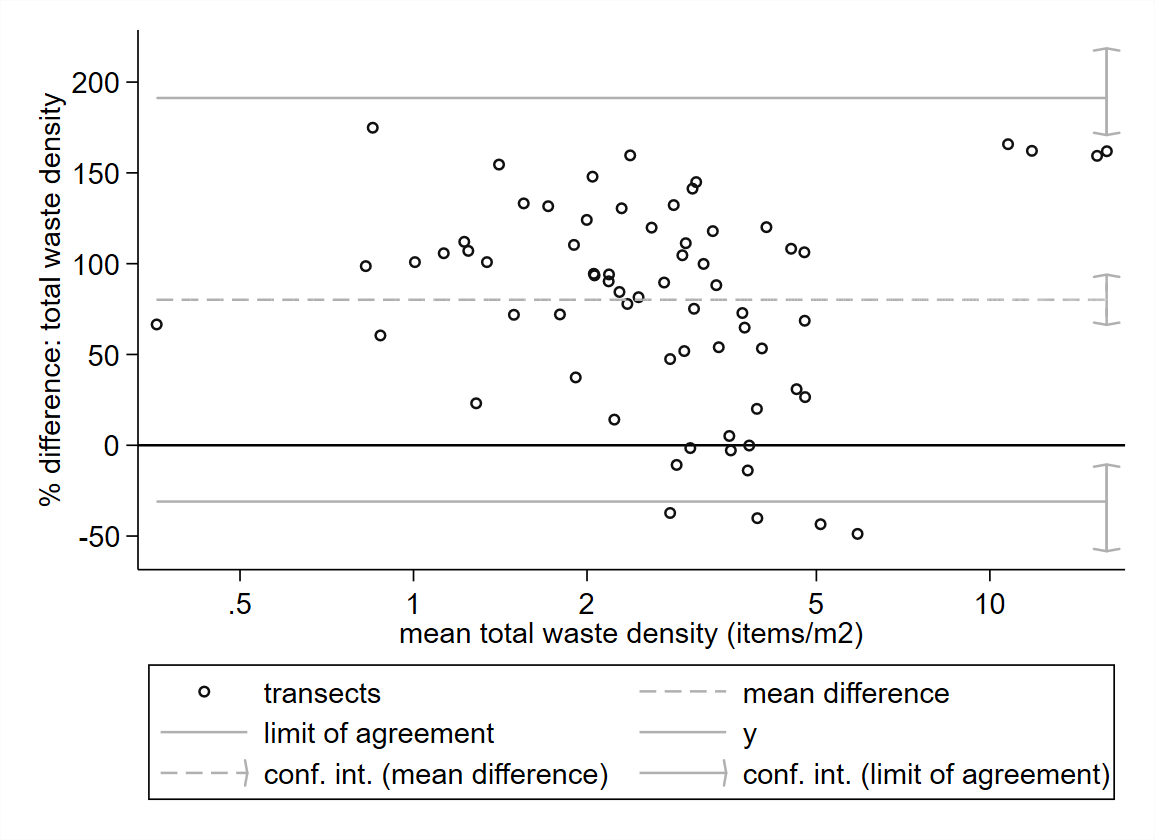

Supplement: Supplementary file 10 — Supplementary file10 (TIF 142 KB) [file 13762_2024_5625_MOESM10_ESM.tif]

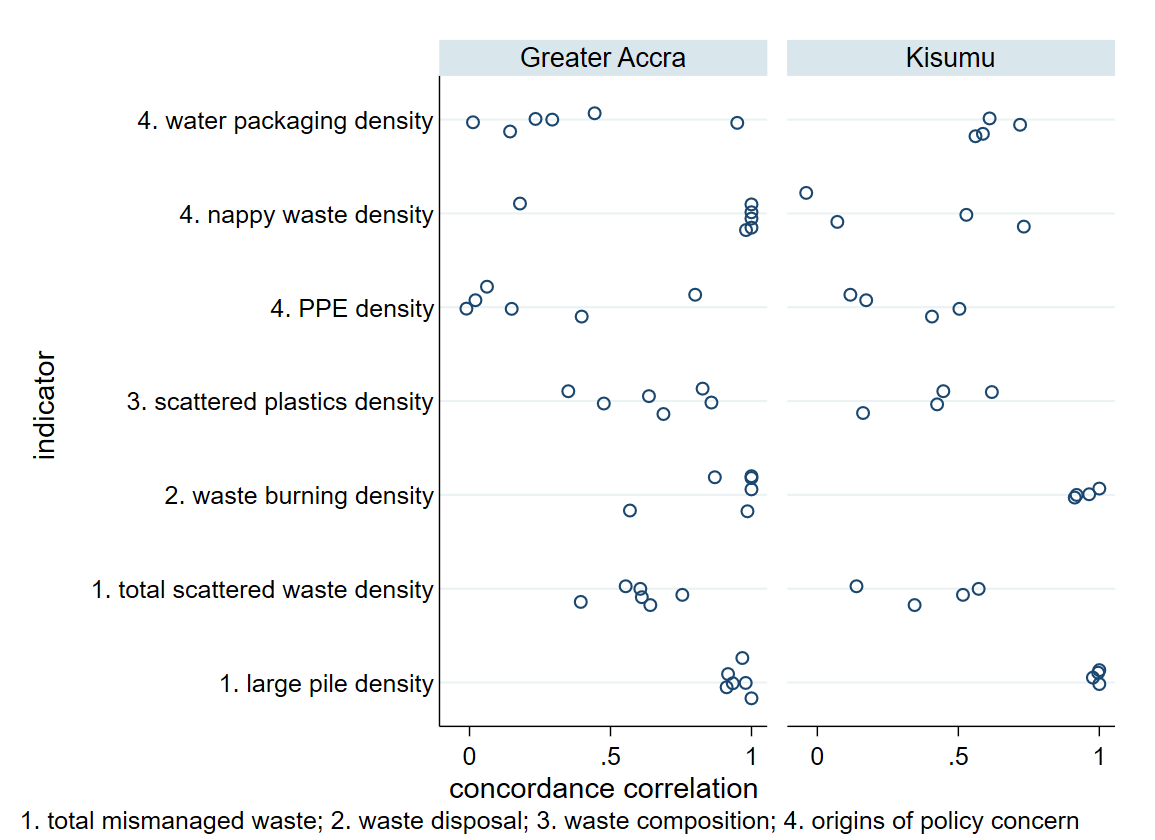

Supplement: Supplementary file 11 — Supplementary file11 (TIF 162 KB) [file 13762_2024_5625_MOESM11_ESM.tif]

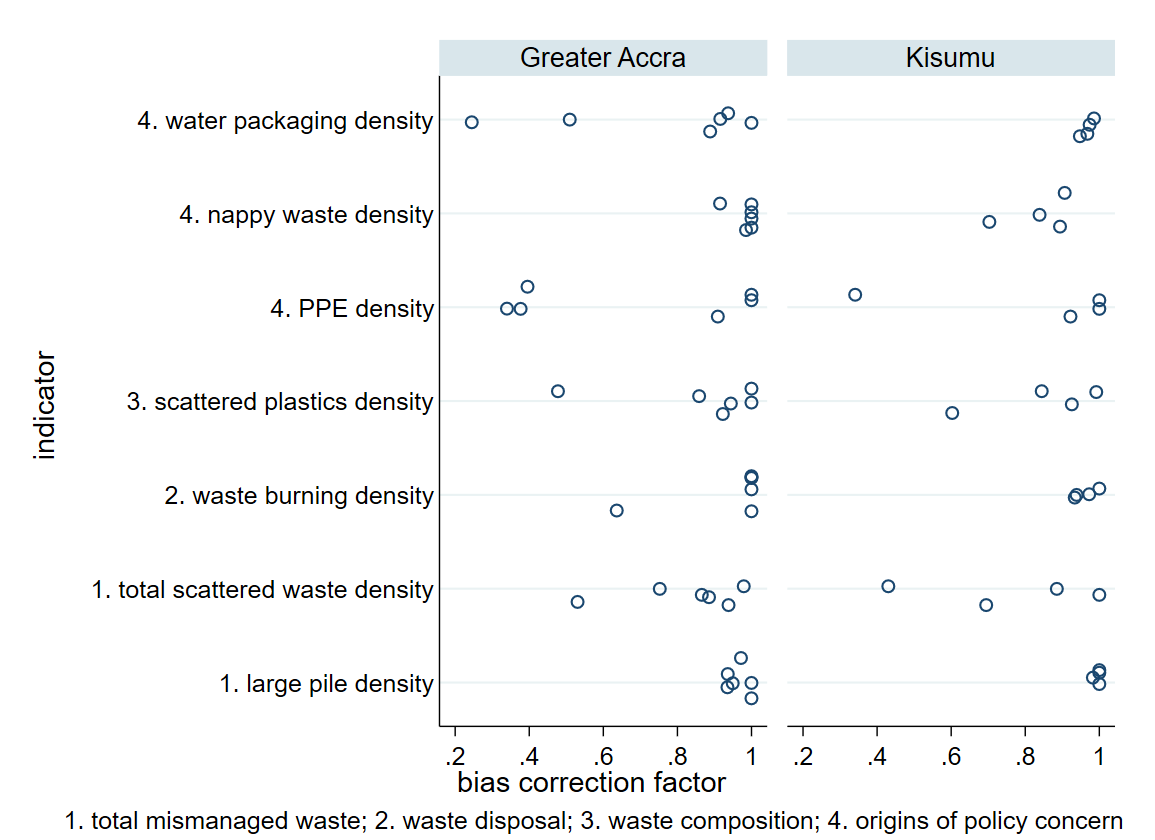

Supplement: Supplementary file 12 — Supplementary file12 (TIF 162 KB) [file 13762_2024_5625_MOESM12_ESM.tif]

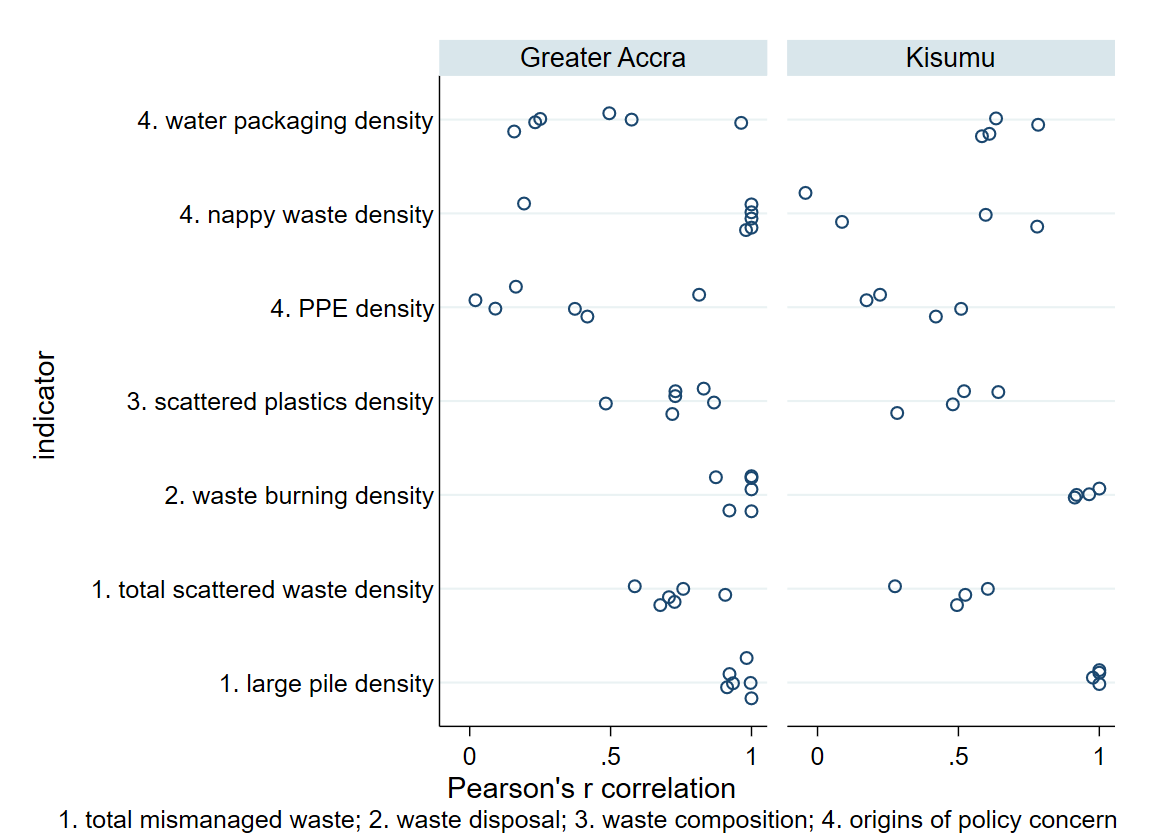

Supplement: Supplementary file 13 — Supplementary file13 (TIF 161 KB) [file 13762_2024_5625_MOESM13_ESM.tif]
