# Supplementary material for: Inter-observer reliability in transect-based observations of environmental waste in greater accra and kisumu: implications for waste management
Source: Int J Environ Sci Technol (Tehran). 2024 Apr 22;21(15):9409–24. doi: 10.1007/s13762-024-05625-5 (PMC11480156; doi:10.1007/s13762-024-05625-5)
Supplement: Supplementary file 14 — Supplementary file14 (DOCX 93 KB) [file 13762_2024_5625_MOESM14_ESM.docx]

**Fig. S1** Bland and Altman limits of agreement plots for scattered waste density recorded by six pairs of transect surveyors in Greater Accra, Ghana (thick solid line represents perfect agreement; thin solid lines are Bland and Altman limits of agreement; dashed line indicates bias, with 95% confidence intervals for bias and limits of agreement shown on the right)
